# The Metropolitan Asylums Board and Permanent Invalid Children

**Published:** 1915-01-02

**Authors:** 


### THE METROPOLITAN ASYLUMS BOARD AND PERMANENT INVALID CHILDREN.

We have drawn attention in our Notes to the suggestion made by the Lambeth Guardians to the Local Government Board that the Managers of the Metropolitan Asylums Board should be authorised to provide accommodation for children likely to become chronic invalids. To a certain extent the Managers already make this provision, as they receive at their various homes and children's hospitals cases of tubercular disease, heart disease, paralysis, and other cases of a chronic character necessitating treatment over long periods of time. The cases the Guardians probably have in mind, however, are those of permanent invalidity not always requiring active medical treatment, but of such a character that they cannot be educated with healthy children. Provision is made for many of these cases in the special schools of the London County Council and in charitable institutions. But there remain many cases which, owing to the nature of their incapacity or to the poverty of their parents, have to be admitted to and kept in the Poor-Law infirmaries. This arrangement leaves much to be desired, because, although they receive here all the medical and nursing attention they need, it is difficult, and often impossible, to arrange for their systematic education, and life in a hospital ward is far from being the best means that could be devised to ameliorate their lot. These children are not ill in the ordinary sense of the word; they are fully capable of appreciating and enjoying the amenities of life, and the necessary restrictions of a hospital are irksome to them. They ought to be in special institutions where they could be educated and taught employments suitable for them, and where they would be free to amuse themselves so far as their physical capacity permitted. It is the duty of the healthy to lighten the load of these burdened lives, to give them so much of the happiness of childhood as is possible for them, and to fit them, so far as may be, to maintain themselves wholly or partially in later life.

The proposal of the Lambeth Guardians has not, however, met with success. The Local Government Board does not consider there is sufficient evidence to justify any action being taken. The Guardians have, therefore, resolved to collect such evidence in the next six months and then to raise the matter again. It is a striking commentary upon the evils arising from the multiplicity of authorities dealing with matters pertaining to the public health in London that there should exist any doubt on the part of the Central Authority whether the number of cases was sufficient to justify special provision being made. Under any scientific system of administration the central office would have on its files, or would be able to obtain at once, a record of every case of the kind chargeable in all the London parishes or known to the school medical officers. No one who has practical knowledge of the London infirmaries can doubt that the number of children in them alone belonging to the class under discussion is amply sufficient to justify the provision of a special institution.

The difficulty as regards the Metropolitan Asylums Board taking any steps in the matter is one of accommodation. The demands upon it during the last few months have been so great that the Board has had to curtail greatly its ordinary admissions. It has had to reduce very considerably the number of sick and convalescent children it usually takes from the infirmaries, and has had to close its doors altogether to cases of measles and whooping-cough. Yet there are scattered throughout the London infirmaries many hundreds of vacant beds, a number which could be still further increased if the medical and nursing staffs were adequate for their work. In truth it is not more institutions that are needed; it is more centralisation, better classification, larger staffs, and fuller equipment. The great defect of the Poor-Law Medical Service is that each unit is trying to deal separately with a mass of heterogenous cases containing a number of small classes which can only be dealt with efficiently in special departments. The problem can be solved only by a combination of units, which will permit the organisation of these departments on a sufficiently large scale for economic working, and will admit of the necessary accommodation being obtained without additional building.

